# Explicit and implicit markers of fairness preeminence in criminal judges

**DOI:** 10.1038/s41598-021-96962-9

**Published:** 2021-09-02

**Authors:** Hernando Santamaría-García, Jorge Martínez Cotrina, Nicolas Florez Torres, Carlos Buitrago, Diego Mauricio Aponte-Canencio, Juan Carlos Caicedo, Pablo Billeke, Carlos Gantiva, Sandra Baez

**Affiliations:** 1 Área Salud, Conocimiento Médico y Sociedad, Centro de Investigaciones sobre dinámica Social. Facultad de Ciencias Sociales y Humanas, Universidad Externado de Colombia, Calle 12 #1-17 Este, 111711 Bogotá, Colombia; 2 Laboratorio de Neurociencia Social y Neuromodulación, Centro de Investigación en Complejidad Social (neuroCICS), Facultad de Gobierno, Universidad del Desarrollo, 7590943 Santiago, Chile; 3 Departamento de Psicología, Universidad de los Andes, Cra. 1 #18a 12, 111711 Bogotá, Colombia

**Keywords:** Psychology, Human behaviour, Social neuroscience

## Abstract

Achieving justice could be considered a complex social decision-making scenario. Despite the relevance of social decisions for legal contexts, these processes have still not been explored for individuals who work as criminal judges dispensing justice. To bridge the gap, we used a complex social decision-making task (Ultimatum game) and tracked a heart rate variability measurement: the square root of the mean squared differences of successive NN intervals (RMSSD) at their baseline (as an implicit measurement that tracks emotion regulation behavior) for criminal judges (n = 24) and a control group (n = 27). Our results revealed that, compared to controls, judges were slower and rejected a bigger proportion of unfair offers. Moreover, the rate of rejections and the reaction times were predicted by higher RMSSD scores for the judges. This study provides evidence about the impact of legal background and expertise in complex social decision-making. Our results contribute to understanding how expertise can shape criminal judges’ social behaviors and pave the way for promising new research into the cognitive and physiological factors associated with social decision-making.

## Introduction

One of the most critical scenarios which criminal judges face is making decisions that have social implications^1–4^. Social decision-making scenarios are strongly modulated by justice, fairness, and reciprocity trends^5^. Despite the importance of social decision-making in tracking justice notions, to the best of our knowledge, no studies have assessed how criminal judges behave in social decision-making scenarios. Additionally, it is unknown to what extent cognitive and physiological predispositions, including executive functioning and resting HRV, could modulate social decisions.

Social decision-making paradigms are useful to determine to what extent individuals fit their behavior to optimize their gains in the presence of others by assessing, updating, and integrating emotional, rewarding, and contextual information^1,4,6–8^. The ultimatum game (UG)^5,8–11^ is a classical task used to track social decision-making. In the UG, a receptor player receives an offer from a proposer and has the option of accepting or rejecting the offer. If the responder rejects the proposer’s offer, neither player is paid. Despite this being against their economic interests, a huge rejection effect of unfair offers has been reported in the UG—an effect considered reciprocal in the presence of injustices^6,12^. Crucially, the UG tracks justice and fairness dispositions and requires strategic thinking, reinforces learning, and uses cognitive control mechanisms to update behavior based on others’ behavior^1,6,13,14^. These processes are of great relevance in legal contexts.

In general, social decision-making is mediated by implicit physiological manifestations that track regulatory mechanisms affecting emotional, cognitive, and arousal processes associated with complex decisions in the presence of others^4,15–18^. One of the most useful techniques to track the implicit physiological manifestations of decision making is the HRV^9^. The resting HRV is defined as a variation in the time interval between heartbeats, and it has been theoretically and empirically shown to indirectly index regulated emotional responses. This measure has served as an indirect trait of individuals’ personality related to ongoing cognitive control and emotional control mechanisms in the presence of mental or psychic stress^19^ and as a biological trait that could predict future decisions^9,20^. Higher resting HRV scores before facing decision-making tasks demonstrates primary cognitive control mechanisms and indicates the offering and rejection rates in bargaining paradigms^21^.

Criminal judges such as judges, classically face difficult decisions as they must assess complex information sources, and they need to integrate all those sources to try to provide fair and justice decisions^2^. Crucially, although previous studies have found that resting HRV in criminal judges could predict moral decision making^2^, no studies have assessed to what extent the resting HRV could track behavior in social decision-making paradigms which require strategic behavior to maintain fairness and reciprocity^5^. Based on this context, in this study, we assessed the behavior of a group of criminal judges (with an average of 14 years (SD = 9.1) of expertise in legal scenarios) conducting a social decision-making task (UG) compared to a control group (individuals without legal expertise). We manipulated different levels of the participants' knowledge on how the proposer considered performing the offerings. In the comparative condition, the participants saw that the proposer assessed two different types of offerings (one of the offerings was fairer than the other). By contrast, in the individual condition, the participant only saw the final offering of the proposer. Using this type of procedure, we want to explicitly manipulate the effect of comparison in the offerings, which is a crucial factor in affecting responses of receptors in economic games^22,23^. Furthermore, we evaluated the resting HRV (i.e., a baseline measurement before performing the UG), a relatively stable individual physiological trait associated with behavior^24^. Resting HRV values can reflect autonomic (parasympathetic) control of the heart, and it is thought to help individuals cope with uncertain and changing environments^24,25^. The resting HRV has been associated with different executive functions and control processes, including cognitive control, emotional regulation, and basal attention before facing tasks or new challenges^26,27^.

The Neurovisceral Integration Model appears to be a crucial framework for understanding the possible associations between the vagally mediated cardiac function indexed by HRV and cognition^28^. According to that model, the HRV, even the resting HRV, is associated with the activity of neural structures responsible for control and regulation, including affective, cognitive, and physiological regulation^29^. Therefore, a greater vagally mediated HRV seems to reflect a good prefrontal neural function, leading to better executive functioning^27^. Thus, the resting HRV is considering a psychophysiological trait evoking behavioral control^30^. Moreover, the resting HRV predicts behavioral control and performance in decision-making scenarios, including UG^31–33^.

Additionally, social decision making is also modulated by a group of executive functions such as planning skills, cognitive control, anticipation, reasoning, and valuing an amount of evidence to reach a decision^34,35^. Particularly, rejecting or accepting ultimatum offers is mediated by individual differences in cognitive control mechanisms. The rejection offers are mediated by intuitive, heuristic-based thinking, whereas accepting offers is related to reflexive, analytics-based thinking^34^. Thus, in our study, we expected significant associations between executive functioning and the rate of rejections and acceptance of offerings in UG in both groups. Crucially, in the criminal judges’ group, we expected that social decisions are mediated by more diverse factors than controls, including biological traits such as HRV, expertise, and cognitive control skills. We hypothesized that criminal judges' fair social decisions would guide their behavior in the UG. Specifically, given the criminal judges' expertise on decision-making on others' transgressions, we expected that their social decisions would promote fair distributions and reject unfair offers more consistently than controls. Moreover, we expected more rejections of unfair offers in the group of criminal judges than in the control group, considering that criminal judges should have more training in detecting and judging injustice and unfair situations. We consider that criminal judges and controls would perform the task differently in the comparative vs. individual conditions. Hypothetically, in comparative rounds the participants would be more severe in rejecting unfair offers, as in this scenario the proposer’ behavior is more explicit. This type of difference should be more pronounced in the criminal judges group. Finally, considering the criminal judges' experience in dealing with complex decisions, we expected significant associations between the resting HRV, an index related to cognitive and emotional control mechanisms, and decisions in UG^26,29,30^. Mainly, we expected significant associations between resting HRV and rejections and acceptance offerings in the criminal judges' group. This effect would be supported by exposition to difficult decisions with legal and social consequences in this group. In addition, this behavior would be supported by significant rejections of unfair offers and slower reaction times than the control group.

## Methods

### Participants

Fifty-one participants took part in this study. The judges included twenty-four subjects who had been criminal judges (mean age = 45.83 yrs., SD = 9.6, M:F 12:12). On average, the judges had 12.13 yrs. of experience in this field (SD = 9.6). Twenty-seven college graduates who did not have a degree in law, did not possess any professional qualification in law, and did not have any work experience related to criminal law were included as a control group (mean age = 42.4 yrs., SD = 11.6). All groups did not differ statistically in terms of age (*p* = 0.265), years of education (*p* = 0.874), sex (chi-squared = 0.62, *p* = 0.431), or executive functioning (*p* = 0.41) (Table 1). All participants were required to declare if they had color perception difficulties. No participant claimed they had this type of alteration.

**Table 1:** Demographic and executive functioning comparisons between groups.

	Criminal judges (*n* = 24)	Controls (*n* = 27)	Judges versus controls
	Mean (SD)	Mean (SD)	*p*-value
**Demographics**			
Age (years)^a^	45.83 (9.6)	42.44 (11.6)	0.265
Sex (M:F)^b^	12:12	16:11	0.431
Education (years)^a^	20.79 (2.9)	19.93 (3.1)	0.874
Experience (years)^a^	12.13 (9.6)	–	–
**Executive functioning**			
IFS total score^a^	25.54 (2.4)	25.02 (2.9)	0.410

^a^*p* values were calculated using independent t-test, ^b^*p* values were calculated using a chi-squared test (*X*^2^). Alpha level set at 0.05. IFS: INECO Frontal Screening battery.

The study was approved by the Ethical Committee of Los Andes University and conducted in accordance with the Declaration of Helsinki. All participants provided informed consent prior to the experimental procedures, as well as relevant information such as socio-demographic data, past job experience, and relevant medical antecedents. In both groups, antecedents of neurological or psychiatric disorders or presence of heart and vascular disease were considered as exclusion criteria. Participants undertook the experiment individually, and, before the games, the researcher spent time promoting a raffle as the participants had to bargain with other players for tokens that they could exchange at the end for raffle tickets.

### Ultimatum game

**Figure 1:**
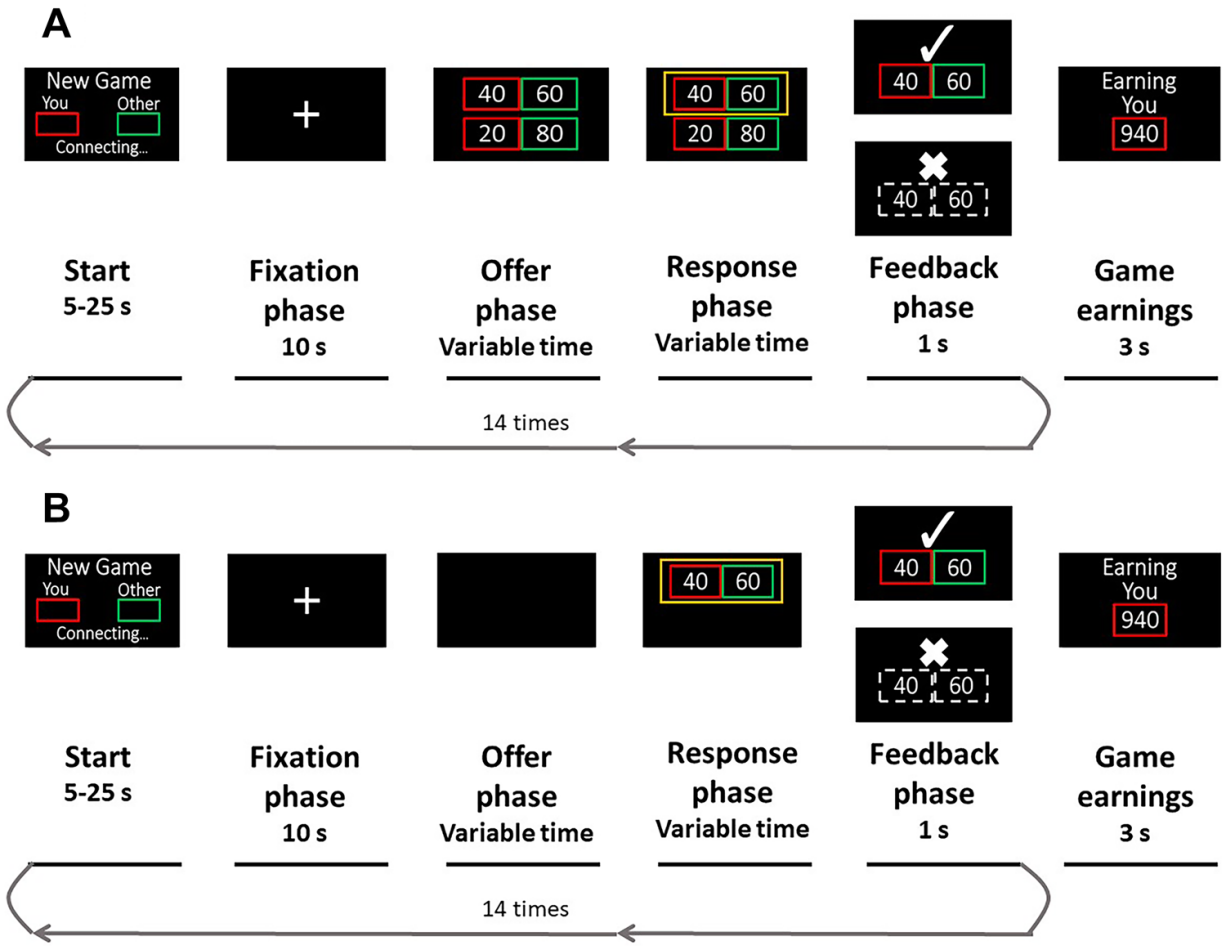
Timeline for the UG. The green box is for the proposer and the red box is for the participants’ role as responders. The trial started by looking at the fixation cross; then, during *comparative condition* (**A**), participants could see the proposer’s options before their final offer (surrounded by a yellow box). During *individual condition* (**B**), participants just could see the final offer. After, responders decided if they would accept and split points or decline and discard points. At the end of each round, the total points earned appear.

We used a modified version of the UG^10,14^, in which participants bargain for a specific resource—in this case tokens that they can later exchange for raffle tickets (each token is worth 10 points). The game consists of two participants, a *proposer*, and a *responder*, who play randomly and anonymously in each round. The proposer suggests a division of points from 0 to 100 (e.g. 60/40: 60 for the proposer, 40 for the responder) between herself and the responder. If the second player accepts, both earn the amount, but if they reject, no one earns points. The game was divided into three phases: (i) an offer phase, when the proposer had to make an offer; (ii) a response phase, when the proposer sent the offer and had to wait for the responder’s answer; and (iii) a feedback phase, when the answer was revealed. At the end of the game, each participant is made aware of their total score (Fig. 1).

All participants played as *responders* and they played a pilot game to become familiar with the setting. Participants were told that they would play with different and anonymous gamers who are playing in other places. The participants played 15 rounds, with two condition types (comparative vs. individual) that were randomly distributed. In the first condition (comparative condition), the participant had two options that the proposer considered in the offer phase (Fig. 1A), while in the second (individual condition), the participant only saw the final offer (Fig. 1B). The rationale for the comparative condition, is to show to the subject the intention underlay the decision-making of the other player in addition to the objective monetary offer given. However, they bargained with the program, which already had a predetermined offer in each trial. The participants played five individual rounds and ten comparative rounds. In total, the participants were offered 40% of fair and 60% of unfair offerings in both comparative and individual rounds. Offers below 30/100 were considered unfair offers, and 50/50 offers were considered fair offers.

Participants saw instructions as follows “You are the RED player. The other player is the GREEN player. The other player must distribute some TOKENS; he/she has two offer options on the screen. The one that he/she chose is highlighted in color. Green for the proposer, red for the other player. Sometimes, you cannot know what the other’s player alternatives are. In those cases, you must decide based on the information you have. You should then accept or reject the offer. If accepted, tokens will be divided; otherwise, if rejected, tokens are discarded, and no one earns tokens in the round”. The measurements obtained were: the rate of acceptance and the rate of rejections in comparative and individual conditions, the rate of rejection of unfair offerings, and the reaction times for each round.

### Heart-rate variability procedure

We used the software LabChart pro version 7.3.7 (ADInstruments, Colorado Springs, CO) to further process and analyze ECG recordings. The aim was to calculate a particular measurement of the beat-to-beat variation of resting HRV (HRV taken during the baseline). The resting HRV measurement has functioned as an indirect trait of individuals’ personality related to ongoing cognitive control and emotional control mechanisms in the presence of mental or psychic stress^19^ and as a biological trait that could predict future decisions^9,20^. In the present study, we used the square root of the mean squared differences of successive NN intervals (RMSSD), and a time-domain component extracted from the HRV, which is considered a vagally mediated measurement of inhibitory control^32^.

The electrocardiogram (ECG) recording was made using the Einthoven lead I configuration with disposable electrodes attached to the left and right wrists. Participants were instructed to relax and close their eyes during ECG monitoring for 10 min. ECG raw data were recorded using a g. USBamp amplifier (sampling rate of 500 Hz).

We first applied various digital FIR filters on the ECG signal to reduce the impact of physiological and technical perturbations. The filters were designed to approximate: (A) a fourth-order low-pass Butterworth filter, (B) a second-order high-pass Butterworth filter, and (C) a notch filter (60 Hz). Subsequently, an algorithm automatically detected the QRS complexes in the recordings, from which R-R intervals were computed (ms). Such automatic identification of R spikes was corroborated by careful visual inspection. If an error was detected, the misplaced marker and the associated R-R intervals were eliminated from the analysis. We removed the segments containing ectopic beats. The resulting R-R time series was used to estimate the RMSSD component. We did not analyze recoding segments that had lost more than 20 percent of the R-R intervals due to artifacts or physiological perturbations.

#### The ineco frontal screening (IFS)

This a sensitive tool used to track cognitive processes associated with frontal functioning^36^. This task has been used in different clinical^37,38^ and non-clinical populations^39^. The IFS is composed by eight subtasks that tracks motor programming, processing of conflicting commands, verbal inhibitory control, the abstraction ability, the backward digit span, the spatial working memory, and a go/no-go test. A mean total score is calculated from the sum of the subtask scores (30 points).

### Procedure

We evaluated the criminal judges in an empty room at their workplaces and the group of controls in an empty room at the Externado University in Bogotá, Colombia. The research group set all the materials, including task sheets, computers, and electrophysiology equipment, in a unique room for two weeks, where we collected all data. Each participant was invited directly from a team member and schedule with different periods avoiding subjects identified between them. Each subject started with an explanation of the informed consent by a research member before a short, structured interview focused on clinical and demographical information. Executive cognitive functioning was assessed after that using the INECO Frontal Screening (IFS). Once the participant finished this, the researcher emphasized that they are going to barge with another online player to obtain tokens for a raffle in the next section. The more tokens he or she obtained, the more opportunities to win.

Further on, a technician connects the electrodes for the EKG and calibrates the equipment before starting with 3 min baseline. After finishing the barging games, the participant was disconnected by the technician. Concluding this period, the participant began the UG task.

### Data analysis

#### Behavioral data analyses

Behavioral data were analyzed using JASP version 0.9.2. All statistical tests used were two-sided. To perform the analyses, we designed a measure that tracks the number of offerings rejected divided by the number of total trials. Thus, we reached a normalized measure of rejections: a rate of rejections. We followed a similar type of analysis with the acceptances of fair offerings and obtained a normalized measure: the rate of acceptance. We assessed the group differences in the UG by employing repeated ANOVA measurements, including the rate of rejection of unfair offers and the type of round (comparative and individual), and the number of trials as within factors, and the group as the between factors. We followed similar procedures to analyze the rate of acceptance of fair offerings. Additionally, we ran a similar type of analysis with the reaction times in rejected trials and similar analysis in accepted trials. We set the significance level at 0.05 for all tests. The generalized eta-squared was used as a measurement of effect size.

To assess the associations between the resting HRV and the UG behaviors (rejections, acceptance, and reaction times in those behaviors) in each group, we ran linear regression models, including as a dependent variable the rate of rejections of unfair offers. As factors, we included the RMSSD measurement, the IFS, the type of rounds, the number of trials, the group, and the interaction between RMSSD × group as predictors. We ran a similar group of analyses but using as a dependent variable the rate of acceptance in fair offerings. Complementary, we also reported the group of correlations (Spearman) between resting HRV and behavior in the UG.

#### Physiological data analysis

Using participants’ ECG recordings, we extracted the low frequency (LF; 0.04 to 0.15 Hz) power component of heart rate variability. We calculated LF power during the baseline period (5 min). We also estimated LF power over several contiguous five-minute recording windows during the task and then computed the average power in this band across the windows. Importantly, groups did not differ in the length of the task recordings (controls: mean duration = 1325.4 s., SD = 306.8; judges: mean duration = 1411.3 s., SD = 313.7; F2, 83 = 0.53, *p* = 0.53). Given that the distribution of LF power was highly skewed, we log-transformed this variable to diminish outlying observation impact.

We extracted a time domain measure, namely the RMSSD following previous procedures^40,41^. The RMSSD is the root mean square of successive differences between normal heartbeats. This value is obtained by first calculating each subsequent time difference between heartbeats in milliseconds. Each of the values is then squared, and the result is averaged before the square root of the total is obtained. The RMSSD reflects the beat-to-beat variance in heart rate and is the primary time domain measurement used to estimate the vagally mediated changes reflected in resting HRV. The RMSSD is correlated with HF power and therefore, also reflects self-regulatory capacity. Arguably, one individual with a significant ability to regulate emotion and arousal who is facing complex decisions should have minor RMSSD scores. A major RMSSD score is positively correlated with the rate of rejection in UG in conventional populations^32,41^.

## Results

A summary of measurements in comparative and individual rounds between groups, including rate of rejections and reactions times, is detailed in Table 2.

**Table 2:** Comparisons between groups in UG rounds and in HRV measurements.

	Criminal judges (mean, SD)	Controls (mean, SD)	Judges vs. controls (*p* value)
**Heart rate variability measurement before UG (resting HRV)**			
RMSSD	16,739 (3988)	75,096 (3548)	< 0.05
**Ultimatum game measures**			
Rate of rejection of unfair offers in individual rounds	0.46 (0.26)	0.39 (0.23)	0.32
Reaction times in individual rounds with unfair offers	5932.22 (4189.2)	4061.62 (2844.22)	0.066
Rate of rejection of unfair offers in comparative rounds	0.78 (0.20)	0.54 (0.40)	< 0.01
Reaction time in comparative rounds with unfair offers	6382.21 (4480.3)	4103.31 (3046.12)	< 0.05
Rate of acceptance of fair offers in Individual rounds	0.515 (0.20)	0.641 (0.27)	0.17
Reaction times in individual rounds with fair offers	6520.11 (4048.91)	3869.2 (2044.40)	0.011
Rate of acceptance of fair offers in comparative rounds	0.719 (0.20)	0.756 (.40)	0.53
Reaction time in comparative rounds with fair offers	5709.11 (4792.12)	4033.84 (4386.01)	0.45

### Behavioral measurements

#### Rejections of unfair offerings

**Figure 2:**
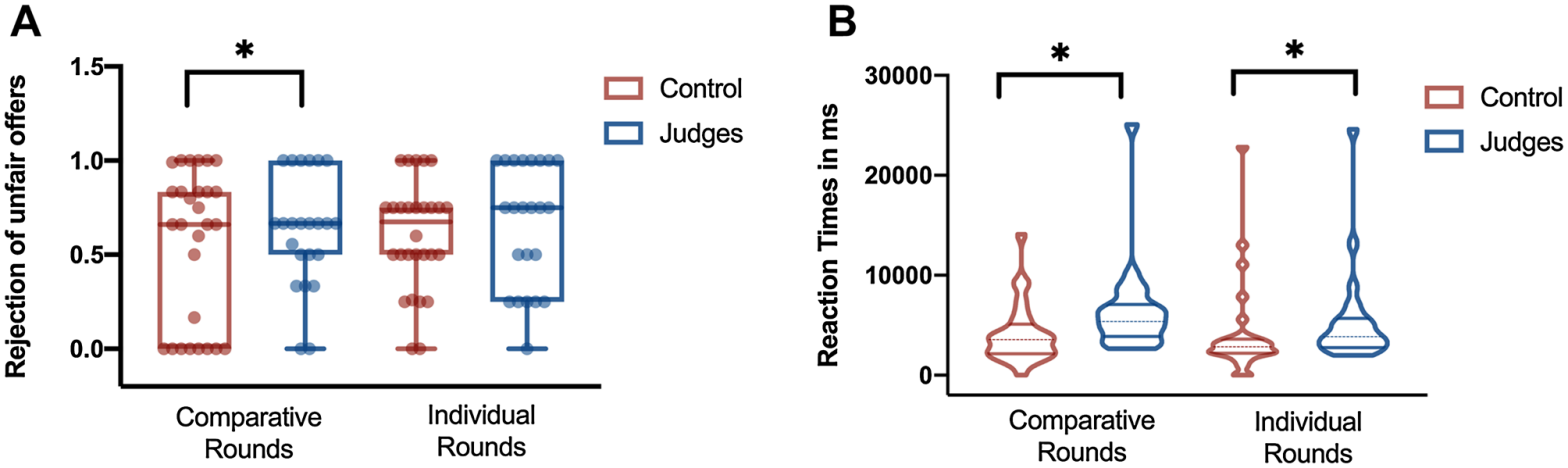
Panel (**A**) shows a group comparison of rejection of unfair offerings in comparative and individual rounds. Panel (**B**) exhibits a group comparison of reaction times in rejected trials between rounds. An Asterix reveals significant group comparisons (*p* < 0.05).

A repeated ANOVA measurement showed a main effect of group [F(1, 46) = 2.11, *p* < 0.05, η2 = 0.03] as judges had a larger rate of rejection of unfair offerings than the control group (*p* < 0.05). We found no interactions between rejections vs. type of round. No other interactions reached significant values (see Table 2 and Fig. 2A). Furthermore, we run a second repeated ANOVA measurement to analyze the reaction times. To balance the analyses of reaction times, we excluded participants that only rejected one trial. Following this criterion, we excluded in these analyses two individuals in the criminal judges group and two individuals in the control group. This analysis showed a main effect of group as criminal judges were slower than controls [F(1, 46) = 5.91, *P* < 0.05, η2 = 0.09]. No interactions between reactions times in rejections and type of round were found [F(1, 46) = 0.22, *P* = 0.63, η2 = 0.01]. No other interactions reached significant values (see Table 2 and Fig. 2B).

#### Acceptance of unfair offerings

The ANOVA showed an interaction between the rate of acceptance of fair offers and type of rounds and group [F(1, 46) = 2.19, *p* < 0.05, η2 = 0.01]. A posthoc analysis showed that criminal judges had a higher acceptance of fair offerings in comparative rounds vs. individual rounds (p < 0.001). No other interactions reached significant results (see Table 2 and Fig. 2A). Finally, we ran a fourth repeated ANOVA measurement to analyze the reaction times in trials accepted. This analysis showed a main effect of group as criminal judges were slower than controls [F(1, 46) = 4.91, *P* < 0.05, η2 = 0.09]. No interactions between reactions times in acceptance trials and type of round were observed [F(1, 46) = 0.22, *P* = 0.63, η2 = 0.01]. No other interactions reached significant values (see Table 2 and Fig. 2B).

#### The association between HRV and UG performance

**Figure 3:**
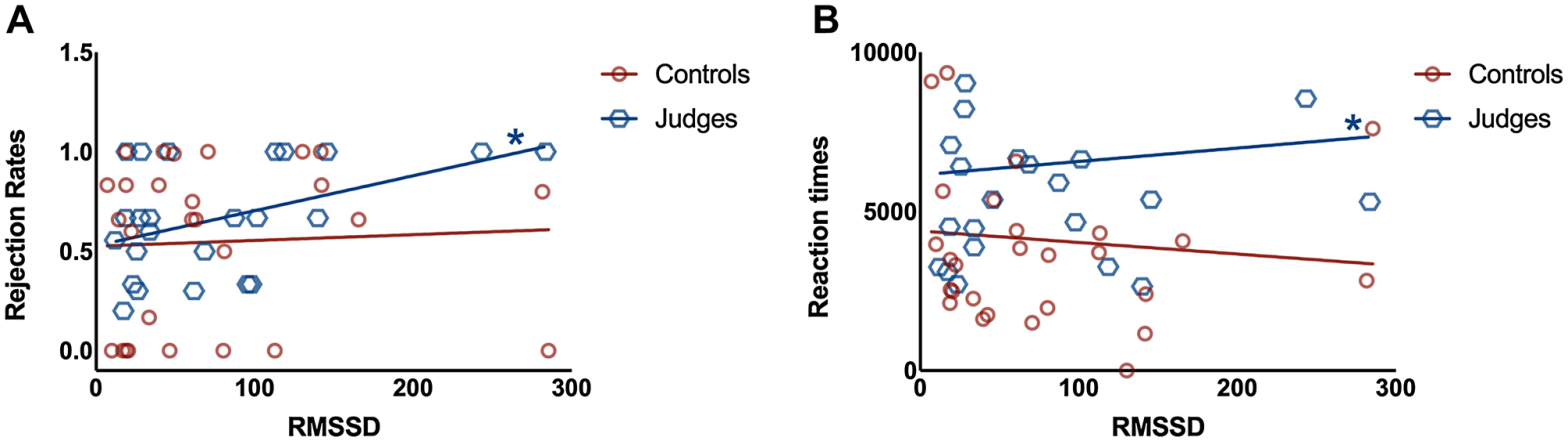
Linear regression models between HRV measurements (RMSSD) and the UG measurements, including the rate of rejections of unfair offers in the comparative rounds, are shown in Panel (**A**); and reaction times in rejected trials are shown in Panel (**B**). An Asterisk reveals significant group comparisons (*p* < 0.05).

We ran a first linear model including the rate of rejection of unfair offerings in the UG as the dependent variable. As factors we included the RMSSD measurement, the IFS, the type of rounds, the number of trials, the group and the interaction between RMSSD x group as predictors. The overall model was statistically significant [F(4, 46) = 2.65, *p* < 0.05, R2 = 0.11]. Analyses of each independent variable showed that group (β = 0.38, t = 2.78, *p* < 0.05) and the RMSSD measurement were significant predictors (β = 0.21, t = 2.14, *p* < 0.05), see Fig. 3. Neither the type of round (β = − 0.01, t = − 0.03, *p* = 0.98), the IFS (β = 0.04, t = 0.31, *p* = 0.76), the number of trials (β = − 0.00, t = − 0.02, *p* = 0.98), the interactions between RMSSD × group (β = − 0.13, t = − 0.33, *p* = 0.73), reached significant values.

Furthermore, to verify the particular associations between the rate of rejections and the resting HRV, we have run correlation analyses of those variables in each group (see Table 3). Those results showed significant associations between the resting HRV and the rate of rejections (r2 = 0.39, *p* < 0.05), and acceptance (r2 = 0.31, *p* < 0.05), in the criminal judges’ group. No associations were observed between the resting HRV and rejections rate (r2 = − 0.231, *p* = 0.23), or acceptance rate (r2 = 0.09, p = 0.62) in control group.

**Table 3:** Correlations between HRV and behavior in Ultimatum game.

Group	HRV measure	Behavioral measures	Pearson	*p* value
Criminal judges	RMSSD	Acceptance of fair offerings in comparative rounds	0.33	0.09
	RMSSD	Rejection of unfair offerings in comparative rounds	0.39	< 0.05
	RMSSD	Acceptance of fair offerings in individual rounds	0.20	0.30
	RMSSD	Rejection of unfair offerings in individual rounds	0.08	0.65
Controls	RMSSD	Acceptance of fair offerings in comparative rounds	− 0.23	0.23
	RMSSD	Rejection of unfair offerings in comparative rounds	− 0.09	0.62
	RMSSD	Acceptance of fair offerings in individual rounds	− 0.20	0.30
	RMSSD	Rejection of unfair offerings in individual rounds	− 0.08	0.65

We ran a similar group of analyzes including the reaction times in the trials rejected in unfair offers. The model included the reaction time in trials rejected as dependent variable and included RMSSD measurement, the IFS, the type of rounds, the number of trials, the group and the interaction between RMSSD × group as predictors. The overall model was statistically significant [F(4, 46) = 2.78, *p* < 0.05, R2 = 0.13]. Analyses of each independent variable showed that RMSSD measurement was a significant predictor (β = − 0.42, t = 2.19, *p* < 0.05). Neither the round (β = 0.07, t = 0.31, *p* = 0.35), the IFS (β = 0.19, t = 1.14, *p* = 0.15), the interaction between RMSSD × group (β = 0.37, t = 1.01, *p* = 0.31), nor the group (β = 0.07, t = 0.36, *p* = 0.71), reached significant values.

A similar model using the rate of acceptance of offerings did not reached significant results [F(4, 46) = 0.30, *p* = 0.87, R2 = 0.02]. We included the reaction time in trials accepted using the same predictors described in previous models in an extra model. The overall model was statistically no significant [F(4, 46) = 0.80, *p* = 0.53, R2 = 0.06] (see Fig. 3).

## Discussion

Ensuring justice requires making complex decisions that have an impact on social scenarios. In this paper, we assessed the social decision-making of individuals who dispense justice as criminal judges and analyzed to what extent physiological predispositions could modulate their social decisions. To the best of our knowledge, the present study is the first experimental attempt at revealing social decision-making in criminal judges. Crucially, our results showed that in complex bargaining scenarios, judges tend to more frequently reject unfair offers than controls, particularly in comparative rounds in which participants testified whether they received the fairest offer between two options (see Table 2).

The UG results revealed that in comparative rounds, judges rejected a significantly higher proportion of unfair offers than the control group. Our results are aligned with previous studies showing a consistent rejection of unfair proposals in humans^12^ and even in non-human primates^42^. However, our results transcend this evidence by showing that expertise and involvement in complex decision making, including those experienced by criminal judges, could modify the rate of rejection of offers based on fairness.

The analyses revealed that the resting HRV was associated with rejection of unfair offerings only in the criminal judges’ group, as indicated by regression and correlation analyses. This pattern of results could suggest that cognitive control mechanisms that are indexed by resting HRV determined the rejections of unfair offerings in the criminal judges’ group. Furthermore, this pattern of results sheds light on the potential role of cognitive control in the criminal judges’ group in overriding cognitive, emotional, and physiological factors that may bias human decisions. This type of explanation coincided with the Neurovisceral Integration Model, which suggests that the HRV can be related to the neurocognitive process underlying cognitive control mechanisms^27^. Differences in rejection rates between groups could be explained by expertise and judges’ exposure to social bargaining scenarios, including when they impart justice. Previous studies have revealed that beyond inter-individual differences in stressful and emotional experiences, UG rejection rates could be modulated by new and continuous stress^12,43^. Moreover, the resting HRV has also shown sensitivity to expertise and exposition factors^20^. Judges could exhibit resting HRV modulations mediated by top-down mechanisms that rely on exposure to stressful situations and expertise in making complex decisions^44,45^. Expertise could promote more regulation of arousal and emotional mechanisms, which allow criminal judges to reject unfair scenarios.

Crucially, the RMSSD has been shown to be related to regulating emotions and behavior mechanisms, a group of processes presumably more stressed in comparative rounds considering that those scenarios call participants to assess between two options if proposer offered the fairest offer or not. Importantly, this scenario requires significant integration of complex information (two possible offerings) and invites participants to make a considerable effort and assessment of rewards in the context—more than in a straightforward scenario in which only one type of offer is seen (individual rounds)^10,46^. Indeed, in comparative rounds, subjects have to integrate the intentions underlying the behavior of others. Knowledge of others' intentions changes decision making despite no differences in rewards across options. This pattern of result aligns with previous studies showing that implicit physiological measurements in complex decisions could determine cognitive control mechanisms and integration of complex information in complex decision-making scenarios^32,47^.

A significant rejection of unfair offers by criminal judges could be explained through reciprocity mechanisms^12,13^. Strong reciprocity theorists argue that the limitations of reputation-based reciprocity models can be overcome by assuming that strong reciprocators who stabilize cooperation by punishing non-cooperators are present within any given community. A strong reciprocator is defined as an individual who is willing to "sacrifice resources for rewarding fair and punishing unfair behavior, even if this is costly and provides neither present nor future material rewards for the reciprocator." This effect has been also called altruistic punishment^48,49^. However, we consider that the pattern of results of our study is more related to the type of activity that criminal judges do routinely. In our concept, the kind of behavior of criminal judges is more mediated by a trend to prefer and decide for fairness than mobilized by strong reciprocity type of punishment. Judges probably behaved by following their expertise in making complex decisions and imparting justice. Moreover, their behavior could be a method of persuading and informing ways to penalize people who propose unfair behaviors. Criminal judges may even be acting as leaders or promotors of fairness as their behavior revealed which offers were actually accepted. In bargaining situations, in particular in there party games, some individuals could assume the role of promotor of justice, and this behavior could implicitly promote fairness in other individuals^5^. Future studies should assess the influence of strong reciprocity in criminal judges' decisions by manipulating another kind of situation in which their own resources are at risk.

Crucially, our results also revealed differences according to the type of round in the rate of acceptance of the fair offerings in the criminal judges' group. Criminal judges accepted more fair offers in comparative trials than in individual trials. No type of round effects was observed in the rate of rejections of acceptance of offerings in the control group. These results suggest that besides rejecting more unfair offerings than controls, the criminal judges differently assess fairness according to the situation. Probably, criminal judges consider that in comparative rounds, the justice was more explicit than in individual rounds. In individual trials, the type of offers was hidden, and the participant might have had doubts about whether there was even a better option to offer. In this case, the judges were able to act on a principle of assuming that fairness was only evident in the comparative trials and therefore accepted fewer offers in the more veiled settings as in the individual rounds. By contrast, the criminal judges had more significant rejected rates than controls regarding unfair offerings, and results revealed that this pattern was unaffected by the type of round. This pattern of results could suggest that criminal judges were highly drastic in assessing unfair offerings irrespective of whether offers were made in comparative or individual scenarios. Future studies could manipulate the autonomy of the proposer to make fair or unfair offerings. In this case, in some trials, the proposer could create an unfair offering by the imposition of the experimental procedure. This type of procedure could simulate real-life situations in which criminal judges must assess if individuals make decisions independently or by third-party impositions.

Our findings suggest that academic background and criminal judges’ expertise seem to shape bargaining behavior. Moreover, the resting HRV seems to be an essential physiological trait that tracks behavior in complex social decisions. Our study empirically supports^50–55^ claims that bodily traits and cognitive and emotional regulation impact social decision-making in individuals highly exposed to complex social decisions. Future studies should assess the extent to which the resting HRV could predict behavior in other social scenarios. Usually, criminal judges must decide on justice in situations where others are involved rather than themselves.

Finally, our findings may have important implications in legal scenarios. Bargaining behavior is a fundamental component of human cultures, which serves to enforce social norms^56^. Indeed, social decision-making is implicitly associated with notions of justice, fairness, and rights^11,56^. Law also has critical regulatory relevance for social life^57^. The legal system must address the sources of bias of defendants, jurors, attorneys, and judges^58^. Our results provide unique evidence by revealing that judges could penalize unfair actions even in in-game scenarios. In other words, judges could transfer their working behaviors to conventional behaviors—for example those in social bargaining games. Usually, criminal judges must decide on justice in situations where others are involved rather than themselves. Thus, future studies should assess the criminal judges' behavior in third-party economic games. Moreover, future studies could assess the extent to which other factors and information on third parties could modulate the criminal judges' behavior.

Although our results could reveal the impact of expertise affecting behavior in UG and matching physiological responses to the behavior in that task, we could consider another explanation. Particularly, certain populations with specific psychophysiological traits (e.g., resting HRV) may be more attracted and have more tendency to endure in the legal sector. Crucially, if this option is possible, our results could be revealing the classical behavioral and physiological profile of individuals who tend to work in legal scenarios. Together, independently of the directionality of the associations, our results revealed a particular form of facing complex social decisions, which are mediated by physiological responses in the group of criminal judges. Future studies should assess the extent to which the disciplinary expertise could modulate the social decisions and the associated physiological responses with longitudinal designs.

Our study has some limitations. First the sample size of our work was relatively small. However, it proved similar to previous studies on social decision-making research in different contexts, including studies assessing UG behaviors^59,60^ and examinations assessing the resting HRV associated with cognitive tasks^32,33^. Future studies assessing the interactions between UG and HRV should include larger sample sizes.

Second, we did not assess the HRV during our study's task (UG). Considering the procedure followed in our research, we only could propose some explanations and indirect inferences about the relationship between HRV, cognitive control, and UG. Although we were interested in analyzing the resting HRV as it is considered an index of basal cognitive control, future studies should assess the HRV when participants perform the task and track the extent to which other cognitive factors could mediate this physiological response. Notably, future studies should determine the interactions between resting HRV, HRV during the task, cognitive control mechanisms, and performance in UG. Furthermore, our study did not include self-report measures associated with changes captured by resting HRV. Therefore, future studies should assess subjective reports to better infer the possible cognitive and emotional processes associated with the physiological activation.

Furthermore, previous studies have discussed the possible limitations associated with response times in the ultimatum game^35,59,61,62^. On the one side, implementing time pressure conditions could affect participants’ behavior and induce selection bias. On the other side, allowing participants to do the task without time restrictions leads to different behaviors, including behaviors promoted by social desirability or strategic-reflexive behaviors (usually named system 2), diminishing the option of capturing the automatic behaviors of participants (traditionally called system 1)^62^. Moreover, some participants could unfollow the dynamics of the paradigm, reducing their engagement in the task. Our study invited participants to answer as fast as possible, but we did not include a time restriction. This procedure could generate substantial dispersion in response times and open the option of some participants to reduce their task’ engagement. Even some studies discarded the information of participants who exhibit considerable reaction times^4,5^. In our research, we have followed a procedure without time restrictions, and we have included all participants in analyzes following the previous procedures^35,63,64^. Future studies should assess social decision-making in individuals with legal expertise by manipulating context, including time pressure, or inducing cognitive load, promoting more automatic and implicit behaviors.

In conclusion, this study provides evidence about the interactions between job type, complex social decision-making, and their physiological and cognitive correlates. We found that criminal judges reject, to a much greater extent, unfair offers than the controls. Furthermore, the criminal judges accept more fair offerings in comparative rounds in which the presumed proposer intentions are more explicit. Moreover, this behavior associated with resting HRV. Our results contribute to understanding the interplay between job type and the cognitive and physiological procedures that subsume complex social decision-making. Crucially, our results pave the way for promising new research into the cognitive and physiological factors associated with legal decision-making.

## Data Availability

The datasets generated are available upon request from the corresponding author.
